# The long-term outcomes of preterm infants receiving non-invasive high-frequency oscillatory ventilation

**DOI:** 10.3389/fped.2022.865057

**Published:** 2022-07-22

**Authors:** Yan Li, Yan Mo, Liping Yao, Qiufen Wei, Danhua Meng, Wei Tan, Xinnian Pan

**Affiliations:** ^1^Maternal and Child Health Hospital of Guangxi Zhuang Autonomous Region, Nanning, China; ^2^Guangxi Clinical Research Center for Pediatric Disease, Nanning, China

**Keywords:** preterm infant, non-invasive ventilation, follow-up, non-invasive high-frequency oscillatory ventilation, outcome

## Abstract

**Objective:**

To investigate the clinical outcomes of preterm infants who received non-invasive high-frequency oscillatory ventilation following extubation in a neonatal intensive care unit.

**Methods:**

Infants born between 25 and 34 weeks of gestation with a birth weight of <1,500 g, who were admitted into the neonatal intensive care unit of Guangxi Maternal and Child Health Hospital, Nanning, Guangxi, China, requiring mechanical ventilation on admission were randomized to the non-invasive high-frequency ventilation group, nasal intermittent positive pressure ventilation group, or nasal continuous positive airway pressure group following extubation. Their respiratory and neurodevelopmental outcomes were assessed at 12 and 24 months of corrected age.

**Results:**

Among 149 preterm infants who underwent randomization, 139 completed their treatment in the neonatal intensive care unit (45, 47, 47 in the non-invasive high-frequency ventilation group, nasal intermittent positive pressure ventilation group, or nasal continuous positive airway pressure group, respectively), 113 were assessed at 12-month corrected age, and 110 of 113 were assessed again at 24-month corrected age. There were no differences in the number of times bronchitis, pneumonia, wheezing episodes, and re-hospitalization rates appeared due to respiratory diseases among the three groups (*P* > 0.05); the pulmonary function tests at 12-month corrected age showed respiratory rate, tidal volume, inspiratory time/expiratory time, time to peak expiratory flow/expiratory time, volume at peak expiratory flow/expiratory volume, expiratory flow at 25, 50, and 75% tidal volume were all similar among infants from the 3 groups (*P* > 0.05). There were no differences in the rates of neurodevelopmental impairment among the three groups at 24-month corrected age (*P* > 0.05).

**Conclusion:**

As post-extubation respiratory support in preterm infants, non-invasive high-frequency ventilation did not increase the rates of long-term respiratory morbidities and neurodevelopmental impairment compared with nasal intermittent positive pressure ventilation and nasal continuous positive airway pressure.

## Introduction

Preterm infants are prone to various conditions because of the immaturity of their organs. Respiratory immaturity is a leading cause of mortality and morbidity in preterm infants. With the advance in neonatal intensive care, particularly the innovation of assistant respiratory support technology, the survival rate of preterm infants improved significantly in the last decades. However, the respiratory complications related to mechanical ventilation (MV), for example, bronchopulmonary dysplasia (BPD), or ventilation-associated pneumonia (VAP), have affected the long-term living quality of preterm infants to a certain degree (1). Non-invasive ventilation is an alternative to MV and has been recommended by American and European guidelines (2, 3). Nevertheless, in preterm infants with moderate to severe respiratory failure, invasive MV was still inevitable. It was reported that 43–80% of preterm infants with respiratory distress received MV (4). Studies on the comparison among non-invasive high-frequency oscillatory ventilation (NHFOV), nasal intermittent positive pressure ventilation (NIPPV), and nasal continuous positive airway pressure (NCPAP) found that NHFOV was superior in infants with difficulties in extubation, such as infants with severe BPD. However, few studies can be found regarding the long-term safety of NHFOV in preterm infants. The objective of this follow-up study is to further explore the safety of NHFOV.

## Materials and methods

### Participants

We conducted a randomized controlled trial (RCT) that compared the short-term outcomes of preterm infants who received NHFOV, NIPPV, or NCPAP as post-extubation respiratory support (Study registered,^1^ trial ID: ChiCTR1900024289) (5). This was a *post-hoc* analysis of the original trial. Infants born with gestational age (GA) of 25 + 0 weeks to 33 + 6 weeks and with a birth weight of less than 1,500 g met the inclusion criteria. A total of 149 eligible preterm infants who required invasive mechanical ventilation on admission and were hospitalized at Guangxi Maternal and Child Health Hospital between 1 April 2017 and 31 October 2018 were included. Non-invasive respiratory support was applied according to randomization. Only the first extubation was analyzed. A total of 149 infants underwent randomization, 139 of 149 completed the study and were discharged from the neonatal intensive care unit (NICU). The surviving infants in the RCT were invited to take part in the followed-up study at 12- and 24-month corrected age (CA).

This study was approved by the Medical Ethics Committee of Guangxi Maternal and Child Health Hospital on March 30, 2017, in Nanning, Guangxi, China ([2017] MESA [5-1]).

### Outcomes

Parents were approached at 12- and 24-month CA by phone interview. Parents were asked to fetch their children’s medical records, and information required by interviewers was extracted from them. Data regarding respiratory diseases, including bronchitis, pneumonia, wheezing episodes, and hospitalization due to respiratory diseases, were collected from parents.

Pulmonary function tests were performed at 12-month CA. Master Screen Paed pulmonary function testing system (Jaeger Germany) was applied. Bronchodilator was discontinued 12 h before testing to avoid the influence caused by medication. Children were in a sleep state, were placed in the supine position, with heads kept at neutral, and with face masks covering their faces and noses. The airflow sensor would detect the parameter while breathing peacefully. The accuracy of volume determination is >0.1 ml, and the accuracy of flow measurement was >0.5 ml/s. The testing would be repeated 5 times consecutively, 20 tidal breathes were recorded each time, and the average value would be calculated automatically by computer. Parameters detected included the following: respiratory rate (RR), tidal volume (VT), inspiratory time/expiratory time (TI/TE), time to peak expiratory flow/expiratory time (TPEF/TE), volume at peak expiratory flow/expiratory volume (VPEF/VE), and expiratory flow at 25, 50, and 75% tidal volume (TEF25%, TEF50%, TEF75%).

Neurodevelopment was assessed by rehabilitation physicians during follow-up visits at 24-month CA at the follow-up clinic in Guangxi Maternal and Child Health Hospital. Neurodevelopmental impairment refers to any of the following occurrences: cerebral palsy (CP), motor or cognition delay, and language delay (6). CP is a disorder of motor development and posture, which causes activity limitations that are due to a non-progressive disturbance occurring in the developing brain. The severity of CP was categorized based on the gross motor function classification system (GMFCS) (7). Motor and cognition development was measured with Gesell Development Scale (GDS), Developmental quotient (DQ) below 75 was considered a motor and cognition delay. Language delay refers to language development that does not achieve the level corresponding to their age (8), and it was assessed with Sign - Significate (S - S) relations.

The primary outcome was neurodevelopmental impairment at 24-months CA (including CP, motor or cognition delay, and language delay). The secondary outcomes were pulmonary function tests performed at 12-month CA, and respiratory diseases occurred within 12- and 24-month CA.

### Statistics

Data were analyzed using the IBM SPSS 22.0 software (IBM Corp., Armonk, NY, United States). Measurement data with a normal distribution are expressed as mean ± SD. A comparison of multiple means was performed by analysis of variance and a two-two comparison was performed by the least significant difference method. Measurement data that were not normally distributed are expressed by the median (interquartile range) and were analyzed by the Kruska–Wallis test. Categorical variables were analyzed by the chi-square or Fisher’s exact test. A *p*-value of <0.05 was regarded as statistically significant.

## Results

**FIGURE 1:**
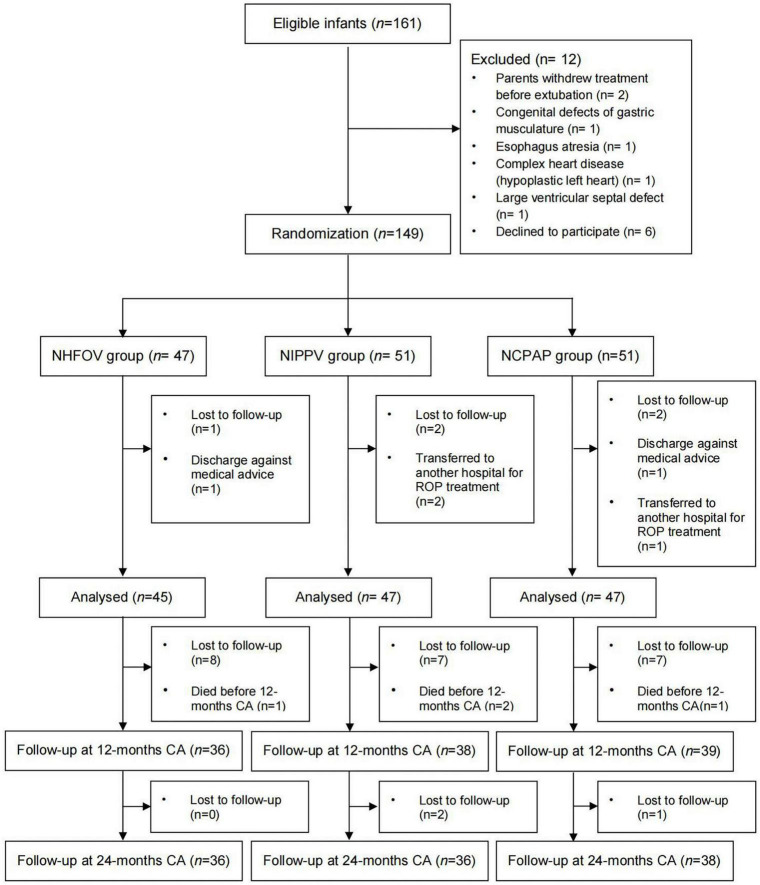
Diagram showing the flow of participants through each stage of the study. NHFOV, non-invasive high-frequency oscillatory ventilation; NIPPV, nasal intermittent positive pressure ventilation; NCPAP, nasal continuous positive airway pressure; CA, corrected age.

Of the 139 infants who completed the study, 4 died before 12-month CA (1, 2, and 1 from the NHFOV, NIPPV, and NCPAP group, respectively), 22 lost to follow-up at 12-month CA, and 113 were assessed (36, 38, and 39 from the NHFOV, NIPPV, and NCPAP group, respectively). We continued to follow-up at 24-month CA, 3 were lost to follow-up, and 110 were assessed (36, 36, and 38 from the NHFOV, NIPPV, and NCPAP group, respectively) (Figure 1).

### Respiratory outcomes at 12- and 24-month corrected age

The number of times of bronchitis, pneumonia, wheezing episodes, and hospitalization within 12- and 24 month CA were similar among the 3 groups. (*P* > 0.05) (Table 1).

**TABLE 1:** Respiratory morbidities at 12- and 24-month CA (number of times).

		N	Bronchitis	Pneumonia	wheezing episode	Rehospitalization
12 months CA	NHFOV group	36	19 (52.8%)	9 (25.0%)	6(16.7%)	9 (25.0%)
	NIPPV group	38	21 (55.3%)	11(28.9%)	7(18.4%)	11 (28.9%)
	NCPAP group	39	21(53.8%)	10(25.6%)	6 (15.4%)	11 (28.2%)
	p value		0.977	0.917	0.938	0.922
24 months CA	NHFOV group	36	14 (38.9%)	6 (16.7%)	7(19.4%)	8 (22.2%)
	NIPPV group	36	17 (47.2%)	7 (19.4%)	7(19.4%)	8 (22.2%)
	NCPAP group	38	16 (42.1%)	8 (21.1%)	8(21.1%)	9 (23.7%)
	p value		0.771	0.889	0.980	0.985

CA, corrected age.

### Pulmonary function tests at 12-month follow-up

At 12-month follow-up, 103 of 113 performed pulmonary function tests. RR, VT, TI/TE, TPEF/TE, VPEF/VE, TEF25%, TEF50%, and TEF75% were all similar among infants from the 3 groups. (*P* > 0.05) (Table 2).

**TABLE 2:** Pulmonary functional tests at 12-month corrected age.

	N	RR(times/min)	VT (ml/kg)	TI/TE (%)	TPEF/TE (%)	VPEF/VE (%)	TEF25% (ml/s)	TEF50% (ml/s)	TEF75% (ml/s)
NHFOV group	33	29.8 ± 3.3	7.8 ± 0.7	73.9 ± 7.2	29.7 ± 3.5	30.1 ± 2.3	54.7 ± 7.4	66.9 ± 11.1	73.4 ± 10.6
NIPPV group	34	29.2 ± 2.5	8.0 ± 0.7	74.0 ± 6.4	30.9 ± 3.6	31.0 ± 2.6	56.3 ± 5.4	66.9 ± 9.2	73.1 ± 8.7
NCPAP group	36	28.7 ± 2.1	7.9 ± 0.7	74.3 ± 5.5	31.3 ± 3.6	30.9 ± 2.6	54.8 ± 6.0	67.2 ± 9.6	71.5 ± 8.6
*p*-value		0.208	0.372	0.945	0.137	0.228	0.450	0.915	0.637

RR: respiratory rate; VT: tidal volume; TI: inspiratory time; TE: expiratory time; TPEF: time to peak expiratory flow; TE: expiratory time; VPEF: volume at peak expiratory flow; VE: expiratory volume; TEF 25%: expiratory flow at 25% tidal volume; TEF 50%: expiratory flow at 50% tidal volume; TEF 75%: expiratory flow at 75% tidal volume.

### Neurodevelopmental outcomes at 24-month corrected age

At 24-month follow-up, the incidence of neurodevelopmental impairment was similar among the 3 groups. (*P* > 0.05) (Table 3).

**TABLE 3:** Neurodevelopmental impairment at 24-month CA.

	N	Neurodevelopmental disability N (%)	Neurodevelopment normal N (%)
NHFOV group	36	7 (19.4%)	29 (80.6%)
NIPPV group	36	8(22.2%)	28 (77.8%)
NCPAP group	38	8 (21.1%)	30 (78.9%)
*p*-value		0.959	

## Discussion

In this follow-up study involving infants with a gestational age of fewer than 34 weeks and a birth weight of less than 1,500 g who received NHFOV, NIPPV, or NCPAP after the first extubation after birth, the mode of non-invasive respiratory support did not affect the respiratory outcomes at 12- and 24-month CA. The mode of non-invasive respiratory support post-extubation did not also affect their neurodevelopmental outcomes at 24-month CA.

Non-invasive high-frequency oscillatory ventilation is a novel mode of non-invasive ventilation that connects non-invasive circuits to high-frequency ventilators, through which gas exchange is achieved by the superposition of high-frequency oscillation over continuous positive flow. Compared with other non-invasive ventilation modes, NHFOV combines the advantages of NCPAP and high-frequency ventilation, making it more effective at maintaining alveolar stability, eliminating CO_2_, and limiting barotrauma (9). Literature has reported that NHFOV reduced the incidence of reintubation in infants with difficulties in extubation due to BPD or other conditions (10, 11). Our trial also found a lower reintubation rate and a lower BPD rate in the NHFOV group (5). Research regarding the safety of NHFOV showed the risks of brain injury, NEC, ROP, and air leak did not increase in preterm infants who received NHFOV, which was compatible with the results of our trial (10, 12). To our acknowledgment, this is the first study reporting the safety of NHFOV from the long-term perspective.

Bronchopulmonary dysplasia (BPD) is the most common respiratory complication in preterm infants. A Chinese multicenter study demonstrated that in preterm infants less than 31 weeks of GA, the incidence of BPD was 12.5%, with a mortality rate of 5.7% (13), survivals are vulnerable to hyperactive airway and recurrent lower-airway infection (14). A previous study of our study group found that the pulmonary function impairment induced by BPD was a predominantly small airway obstruction (15). In the original trial, the duration of non-invasive ventilation differed among the 3 groups (9 vs. 11 vs. 17 days for infants randomized to NHFOV, NIPPV, and NCPAP groups). When followed to 12- and 24-month CA, the number of times bronchitis, pneumonia, wheezing episodes, and rehospitalization was similar among the 3 groups. The pulmonary function tests performed at 12-month CA revealed a trend of small airway obstruction in the study population; however, the results of the tests were similar among the 3 groups. The explanation is that the GA of infants involved in this trial was relatively big, and the respiratory diseases are rather multifactorial, therefore, the impact of the duration of non-invasive respiratory support during the early stage of life on the long-term respiratory morbidities is minor.

Neurodevelopmental impairments play an important role in the long-term living quality of preterm infants. It was reported that invasive HFOV might increase periventricular leukomalacia (PVL) or severe IVH (16, 17). Nevertheless, it is still controversial, because the multicenter study demonstrated that the incidence of PVL or IVH did not increase in infants who received invasive HFOV (18). Research involving the influence of NHFOV on the risks of PVL or IVH is rare (19). In our trial, the incidence of severe IVH (grade III and above) is similar in the NHFOV group compared with the NIPPV group and the NCPAP group (5). When followed for 24-month CA, the neurodevelopmental outcomes remained reassuring in the NHFOV group. The explanation is that the long-term neurodevelopmental outcomes of preterm infants were mainly affected by GA, the presence of severe IVH or PVL, and were related to the social-economic status of parents (20, 21).

The strength of this study is that it is the first follow-up study to compare the respiratory and neurodevelopmental outcomes of preterm infants who received NHFOV with those who were treated with NIPPV or NCPAP. The results enhanced the confidence in the application of NHFOV in clinical practice. There were also limitations of this study. This was a *post-hoc* analysis of the original study. The sample size was small, and the follow-up rate was relatively low. A prospective multi-center study is required to balance the bias caused by other clinical interventions. Studies focusing on specific subgroups, such as extremely preterm infants or infants with severe BPD, may better illustrate the possible advantages of NHFOV. In addition, we were not able to assess the severity of the adverse outcomes from both respiratory and neurodevelopmental perspectives.

## Conclusion

In conclusion, as post-extubation respiratory support preterm infants, non-invasive high-frequency ventilation did not increase the rates of long-term respiratory morbidities and neurodevelopmental impairment compared with nasal intermittent positive pressure ventilation and nasal continuous positive airway pressure.

## Data availability statement

The raw data supporting the conclusions of this article will be made available by the authors, without undue reservation.

## Ethics statement

The studies involving human participants were reviewed and approved by the Medical Ethics Committee of Guangxi Maternal and Child Health Hospital.

## Author contributions

YL, QW, YM, and XP contributed to the conception and design of the study. YL, YM, LY, WT, and DM prepared the materials and performed the data collection and analysis. YL and YM wrote the first draft of the manuscript. All authors provided comments on the manuscript and read and approved the final manuscript.

## Conflict of interest

The authors declare that the research was conducted in the absence of any commercial or financial relationships that could be construed as a potential conflict of interest.

## Publisher’s note

All claims expressed in this article are solely those of the authors and do not necessarily represent those of their affiliated organizations, or those of the publisher, the editors and the reviewers. Any product that may be evaluated in this article, or claim that may be made by its manufacturer, is not guaranteed or endorsed by the publisher.

## Footnotes

1
http://www.chictr.org.cn
