# Supplementary material for: The long-term outcomes of preterm infants receiving non-invasive high-frequency oscillatory ventilation
Source: Front Pediatr. 2022 Jul 22;10:865057. doi: 10.3389/fped.2022.865057 (PMC9353142; doi:10.3389/fped.2022.865057)
Supplement: Supplementary file 1 [file Table_1.docx]

Supplementary table: Baseline characteristics

| **Group** | **NHFOV** | **NIPPV** | **NCPAP** | **P value** |
| --- | --- | --- | --- | --- |
| **N** | 36 | 36 | 38 |  |
| **Male (%) †** | 17 (47.2) | 20 (55.6) | 19 (50.0) | 0.771 |
| **GA (weeks) ‡** | 29.5±1.4 | 29.5±1.3 | 29.4±1.1 | 0.845 |
| **BW (g) ‡** | 1117.8±214.4 | 1106.7±158.7 | 1144.7±205.9 | 0.688 |
| **Antenatal steroids (%) †** | 35 (97.2) | 34 (94.4) | 36 (94.7) | 1.000* |
| **Surfactant (%) †** | 18 (50.0) | 15 (41.7) | 18 (47.4) | 0.769 |
| **CRIB-score (point) ‡** | 7.8±2.4 | 7.7±1.9 | 7.5±2.1 | 0.794 |
| **5-min Apgar score ‡** | 8.5±1.3 | 8.3±1.7 | 8.6±1.4 | 0.635 |
| **Duration of MV (d) †** | 3.0 (1.0, 6.5) | 3.0 (1.0, 9.8) | 3.0 (1.0, 7.0) | 0.592**¶** |
| **Reintubation rate†** | 3 (8.3) ^b^ | 9 (25.0) ^c^ | 21 (55.3) | 0.000 |
| **VAP** | 1 (2.8) | 2 (5.6) | 1 (2.6) | 0.824* |
| **BPD** | 10 (27.8）^b^ | 12 (33.3) ^c^ | 23 (60.5) | 0.009 |
| **NEC**  **(stage Ⅱ and above)** | 2 (5.6) | 1 (2.8) | 3 (7.9) | 0.870* |
| **ROP**  **(stage Ⅱ and above)** | 6 (16.7) | 7 (19.4) | 9 (23.7) | 0.749 |
| **PDA** | 13 (36.1) | 12 (33.3) | 11 (28.9) | 0.803 |
| **IVH**  **(grade Ⅲ and above)** | 1 (2.8) | 2 (5.6) | 1 (2.6) | 0.842* |

***NHFOV:*** *non-invasive high frequency oscillatory ventilation;* ***NIPPV:*** *non-invasive intermittent positive pressure ventilation;* ***NCPAP:*** *nasal continuous positive airway pressure;* ***GA:*** *gestational age;* ***BW:*** *birth weight;* ***MV****: mechanical ventilation;* ***VAP****: ventilation associated pneumonia;* ***BPD****: bronchopulmonary dysplasia;* ***NEC****: necrotizing enterocolitis;* ***ROP****: retinopathy of prematurity;* ***PDA****: patent ductus arteriosus;* ***IVH****: intraventricular hemorrhage.*

**†***Data are medians (interquartile ranges) or frequencies (percentages).*

**‡***Data are means ± standard deviation.*

^¶^ *represented by rank sum test.*

** Fisher exact test.*

^a^ *NHFOV group compare with NIPPV group, p＜0.05*

^b^ *NHFOV group compare with NCPAP group, p＜0.05*

^c^ *NIPPV group compare with NCPAP group, p＜0.05*
